# ImmunoPET Targeting Receptor Tyrosine Kinase: Clinical Applications

**DOI:** 10.3390/cancers15245886

**Published:** 2023-12-18

**Authors:** Flavia Linguanti, Elisabetta Maria Abenavoli, Raffaella Calabretta, Valentina Berti, Egesta Lopci

**Affiliations:** 1 Nuclear Medicine Unit, Department of Experimental and Clinical Biomedical Sciences “Mario Serio”, University of Florence, 50134 Florence, Italy; flavialinguanti@gmail.com (F.L.); valentina.berti@unifi.it (V.B.); 2 Nuclear Medicine Department, Ospedale San Donato, 52100 Arezzo, Italy; 3 Nuclear Medicine Division, Careggi University Hospital, 50134 Florence, Italy; 4 Division of Nuclear Medicine, Department of Biomedical Imaging and Image-Guided Therapy, Medical University of Vienna, 1090 Vienna, Austria; 5 Nuclear Medicine Unit, IRCCS—Humanitas Research Hospital, Via Manzoni 56, 20089 Rozzano, Italy

**Keywords:** ImmunoPET, PET/CT, receptor tyrosine kinases, RTK, EGFR, HER2, HER3, VEGF

## Abstract

**Simple Summary:**

Receptor tyrosine kinases (RTKs) are a family of transmembrane proteins that play crucial roles in regulating various cellular processes. The introduction of ImmunoPET targeting RTKs by specific monoclonal antibodies (mAbs) or antibody fragments is regarded as a promising tool for imaging treatment efficacy and developing anticancer drugs. Herein, we review the current clinical research on ImmunoPET targeting RTKs, with particular interest in the epidermal growth factor family, or HER family, and vascular endothelial-derived growth factor/receptor.

**Abstract:**

Receptor tyrosine kinases, or RTKs, are one large family of cell surface receptors involved in signal transduction, which represent an integral part of the signaling pathways. They play a crucial role in most important cellular processes, starting with the cell cycle, proliferation and differentiation, as well as cell migration, metabolism and survival. The introduction of ImmunoPET evaluating the expression of RTKs by specific monoclonal antibodies (mAbs) or antibody fragments is regarded as a promising tool for imaging treatment efficacy and developing anticancer therapeutics. Our review focuses mainly on the current clinical research regarding ImmunoPET targeting RTKs, with particular interest in the epidermal growth factor family, or HER family, and vascular endothelial-derived growth factor/receptor.

## 1. Introduction

Immunotherapy has revolutionized oncologic treatments, changing the landscape for numerous cancer types [1]. In this context, the implementation of monoclonal antibodies (mAb) targeting tumor-related antigens, such as receptor tyrosine kinases (RTKs), has become the standard of care in many malignancies [2]. Nevertheless, their therapeutic potential has to face several challenges, including the definition of optimal dosing and scheduling, the delayed response kinetics and the lack of predictive biomarkers [3]. Moreover, the selection of patients who could benefit the most from these treatments is still a matter of research. In fact, both immunohistochemistry (IHC) and functional imaging with traditional radiotracers are still inadequate to foresee responders and no-responders and to evidence inter- and intra-patient heterogeneity [4].

**Figure 1:**
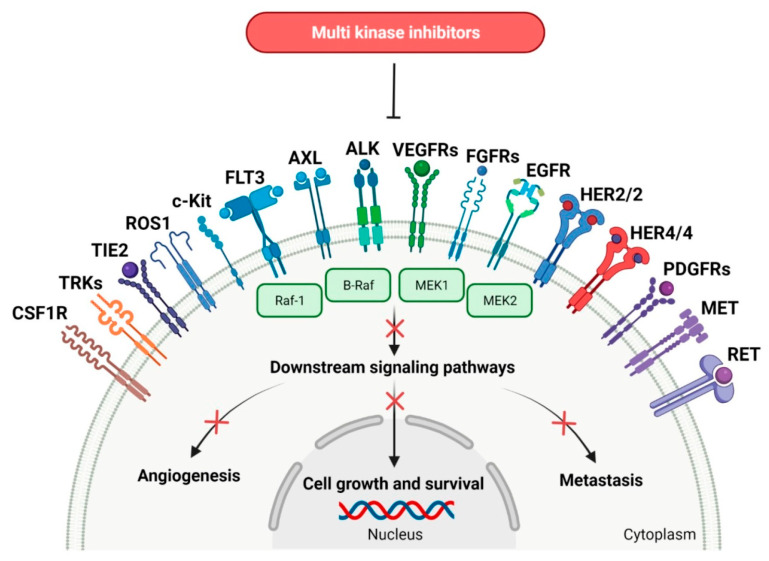
The schematic figure illustrates the sequence of events that occur upon RTK activation (the “x” on the arrows indicates process inhibition): 1. Ligand binding: the specific ligand binds to the extracellular domain of the RTK; 2. Dimerization: the binding of the ligand induces the RTK to dimerize, bringing the intracellular domains of the two RTKs into close proximity; 3. Cross-phosphorylation: tyrosine residues within the intracellular domains of the RTKs are phosphorylated by each other; 4. Activation of downstream signaling pathways: the phosphorylated tyrosine residues serve as docking sites for various signaling proteins, initiating a cascade of downstream signaling pathways; 5. Cellular response: the activation of downstream signaling pathways ultimately leads to cellular responses such as cell proliferation, differentiation, survival and motility. Notes: CSF1R: colony-stimulating factor 1 receptor. TRKs: tropomyosin receptor tyrosine kinases. TIE2: tunica interna endothelial cell kinase 2. ROS1: proto-oncogene tyrosine-protein kinase ROS. c-Kit: mast/stem cell growth factor receptor. FLT3: FMS-like tyrosine kinase-3. AXL: AXL receptor tyrosine kinase. ALK: anaplastic lymphoma kinase. VEGFRs: vascular endothelial growth factor receptors. FGFRs: fibroblast growth factor receptors. EGFR: epidermal growth factor receptor. HER2/2: human epidermal growth factor receptor 2 and 2. HER4/4: human epidermal growth factor receptor 4 and 4. PDGFRs: platelet-derived growth factor receptors. RET: receptor tyrosine kinase rearranged during transfection. B-Raf: serine/threonine-protein kinase B-Raf. Raf-1: RAF serine/threonine-protein kinase. MEK1: mitogen-activated protein kinase 1. MEK2: mitogen-activated protein kinase 2. MET: mesenchymal-epithelial transition factor. Created with BioRender.com and published on https://encyclopedia.pub/entry/22089#ref_67 (last access: 26 November 2023) as adaptation from Sochacka-Ćwikła A et al. [8] under a Creative Commons Attribution 4.0 International License http://creativecommons.org/licenses/by/4.0/ (last access: 26 November 2023).

The introduction of ImmunoPET, or immuno-positron emission tomography, represents a paradigm-shift for the evaluation of the targeting specificity of mAbs [5]. ImmunoPET is a molecular imaging technique that combines the specificity and affinity of antibodies with the sensitivity and resolution of PET imaging [5]. It involves the use of radiolabeled antibodies or their fragments to target specific tumor markers, immune cells, immune checkpoints and inflammatory processes. This allows for non-invasive visualization of these targets in the body, aiding in the diagnosis, staging and monitoring of response to treatment in various cancers [6,7]. This newer imaging could reveal the expression of RTKs (Figure 1), which are one of most explored targets for developing anticancer therapeutic and imaging agents [7,8,9,10,11,12] (Table 1). ImmunoPET has, in fact, shown excellent specificity and sensitivity in detecting tumors expressing RTKs and assessing changes in expression levels in response to targeted kinase inhibition [9]. This technique serves as a functional sensor of plasma membrane levels of RTKs in tumors, providing valuable insights into the response to targeted kinase inhibition [13].

Several isotopes are commonly used in ImmunoPET imaging, each with its own advantages and disadvantages. Fluorine-18 (^18^F) is a popular choice due to its short half-life (110 min) and favorable decay mode, which produces high-energy positrons suitable for PET imaging. Other isotopes used in ImmunoPET include copper-64 (^64^Cu), zirconium-89 (^89^Zr) and gallium-68 (^68^Ga). The choice of isotope depends on the specific application, considering factors such as half-life, decay mode and availability [5].

As for other procedures, ImmunoPET presents several challenges, including not only the identification of the optimal radionuclide for labeling, but also its dosing and cold labeling, the latter one aiming to enhance the imaging specificity. It is also essential to acknowledge potential off-target effects of the molecules utilized. These effects arise from the interaction of the radiotracer or its carrier molecule with non-target tissues. The most common off-target effects include inflammation and radiation exposure [5,10]. Moreover, challenges such as suboptimal imaging properties, the low expression of targets in tumor lesions and high background activity in some organs have been noted. These also depend on whether mAbs or antibody fragments are used and whether full-length antibodies show slow blood clearance, reduced target-to-background ratio and require long-lived radionuclides for labeling. Moreover, drawbacks such as production costs, infrastructure and insurance issues, can limit the use of ImmunoPET to only highly specialized centers and would thus hamper the availability for all countries. Hopefully, ongoing research is addressing these challenges and is expected to improve the technique [5,10].

In the present review we focus on the current clinical research regarding ImmunoPET imaging targeting RTKs, with particular interest in the (HER) family and vascular endothelial-derived growth factor/receptor (VEGF/VEGFR).

## 2. Receptor Tyrosine Kinases (RTKs) Family

Receptor tyrosine kinases (RTKs) are a family of transmembrane proteins that play crucial roles in regulating various cellular processes, including cell growth, differentiation, survival and motility. These proteins are activated by the binding of specific ligands, leading to the phosphorylation of tyrosine residues on their intracellular domains (Figure 1). This phosphorylation triggers a cascade of downstream signaling pathways that ultimately control cellular responses. Dysregulation of RTK signaling is a hallmark of cancer, as it can lead to uncontrolled cell growth, invasion and metastasis. Various mechanisms contribute to RTK dysregulation in cancer, including gene amplification, mutations and the overexpression of RTKs or their ligands. The RTK family encompasses a diverse group of proteins with varying roles in cancer development. Some of the most well-characterized RTKs implicated in cancer include the “HER family”, i.e., epidermal growth factor receptor (EGFR), ErbB2 (neu, HER2), ErbB3 (HER3) and ErbB4 (HER4), as well as VEGF/VEGFR, which is discussed in the following paragraphs [14,15,16,17,18].

### 2.1. EGFR

Epidermal growth factor receptor, or EGFR, is a transmembrane protein that plays a crucial role in regulating cell growth, proliferation, differentiation and survival. It belongs to the ErbB family of RTKs, which are activated by binding to their respective ligands. EGFR is activated by EGF, a growth factor that is involved in various cellular processes, including cell division, migration and adhesion [19]. EGFR consists of an extracellular domain that binds to EGF, a transmembrane domain, and an intracellular domain that contains the tyrosine kinase activity. Upon EGF binding, EGFR undergoes dimerization and autophosphorylation, activating its tyrosine kinase activity. This activation triggers a cascade of downstream signaling pathways that regulate various cellular processes, including the MAPK/ERK (mitogen-activated protein kinases/extracellular signal-regulated kinase) pathway, which regulates cell proliferation and differentiation; the PI3K/AKT (phosphatidylinositol 3-kinase/protein-chinasi B) pathway, which promotes cell survival and growth; and the JAK/STAT (Janus Kinase/Signal Transducer and Activator of Transcription) pathway, which regulates cell proliferation, differentiation and immune response [20,21,22]. EGFR overexpression or mutations are frequently observed in cancer types such as non-small cell lung cancer (NSCLC), head and neck squamous cell carcinoma (HNSCC), colorectal cancer, gastric cancer and breast cancer [23]. This has driven the development of several classes of drugs that specifically target EGFR, including mABs and TKIs, such as cetuximab, panitumomab, erlotinib, gefitinib, afatinib, osimertinib and dacomitinib [21,22].

Cetuximab is a chimeric monoclonal antibody that targets EGFR. It binds to the extracellular domain of EGFR, blocking phosphorylation and activation of receptor-associated kinases (MAPK and PI3K/Akt). This blockade prevents EGFR from initiating downstream signaling pathways that promote cell growth, proliferation and survival. As a result, cetuximab effectively halts the growth and survival of EGFR-dependent cancer cells. [22,24].

The interest in positron-emitting radioisotopes compatible with Cetuximab has led to an increase in use of ^89^Zirconium [^89^Zr]. Considering the biologic half-life of cetuximab in blood equal to 65–95 h and the half-life of [^89^Zr] of approximately 78 h, there is a concrete possibility to perform more scans over time and compare them to each other [25]. The first human study was conducted by Menke-van der Houven van Oordt et al. in a cohort of patients affected by metastatic colorectal cancer (mCRC) [26]. They assessed the optimal timing at 6 days after injection for the visual assessment, as well as to calculate SUVs (standardized uptake values), discriminating the specific uptake versus background activity. They analyzed SUV values in relation with response to cetuximab treatment with positive correlation.

To optimally reflect the biodistribution of cetuximab and to bind physiological/non tumor sites, it is possible to administrate a scouting dose of unlabeled therapy [27]. In the specific case, the liver is often affected by metastasis, but at the same time, it represents a physiologic site of [^89^Zr] uptake. The spill-over phenomenon of healthy hepatic tissue turns the pathological sites unreliable, reducing the detection of pathological lesions.

The possibility to identify cetuximab responders was also assessed in other types of cancers. In fact, Van Loon et al. [28] determined the safety of [^89^Zr]Zr-cetuximab and the assessment of tumor uptake with the highest chance of benefit in patients affected by NSCLC and head and neck cancer (HNC). In this study, the trend of tumor to blood ratio (TBR) was reported as higher at intervals >5 days after injection, but without significant difference in dose schedules (60 mBq + 60 mBq at 0 and at 14 day versus 120 mBq at 0 day). Visual assessment showed rather patchy distribution, without an evident tumor-specific uptake. Finally, they found absence of significant correlation between [^89^Zr]-cetuximab uptake and EGFR at immunohistochemistry (IHC).

The use of cetuximab labeled with [^89^Zr] in locally advanced HNC was also studied by Even et al. [29]. They compared scans performed at either days 3 and 6 or at days 4 and 7 post-injection and confirmed the strong correlation between TBR value and the time of scan, with maximum values at 7 days post-injection due to the reduction of the background activity. An important aspect of this study is the inter-patient variability in TBR values, suggesting the possibility to predict treatment outcome. By dividing patients into two groups according to EGRF expression at IHC, the authors found a significant difference in [^89^Zr]Zr-cetuximab SUV, but no difference in TBR values. So, higher EGFR expression was correlated with higher [^89^Zr]Zr-cetuximab SUVmean and SUVpeak. Finally, they demonstrated a strong correlation for TBR in the pathological lymph nodes and primary tumor. This might indicate that uptake is mainly determined by intrinsic characteristics of the tumor cells.

**Figure 2:**
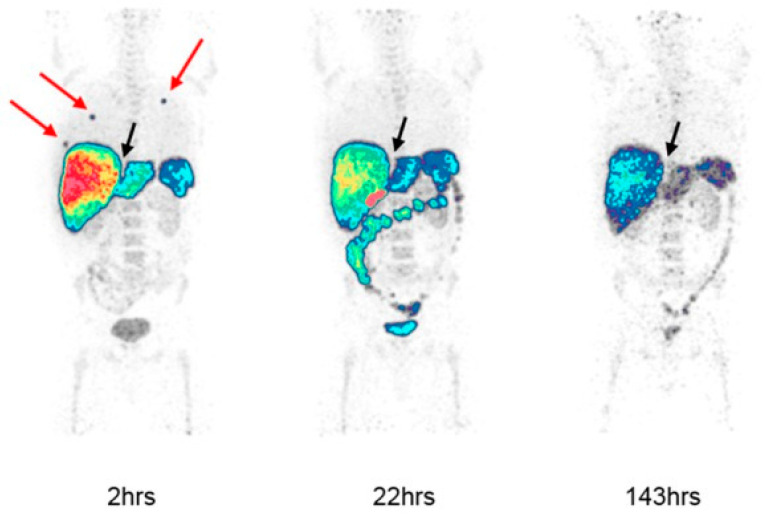
Maximum intensity projection (MIP) images of [^89^Zr]Zr-panitumumab PET acquired at 2, 22 and 143 h in a patient with metastatic colorectal cancer. The focal tracer accumulations (red arrows) in the lungs are noted at 2 h imaging and resolved completely on subsequent scans. Meanwhile, the liver metastases appeared photopenic (black arrow). Note the high physiologic accumulation of the tracer in the liver, spleen, bowels and bladder. Reproduced from Lindenberg L, et al. [31] under “Creative Commons Attribution Noncommercial License” (last access: 26 November 2023).

EGFR expression has also been studied using panitumumab labeled with [^89^Zr]Zr-Panitumumab is a completely humanized mAb that binds to the EGFR, and it is FDA (Food and Drug Administration)-approved for use in receptor expressing colorectal cancers without KRAS mutations [30]. Lindenberg et al. exposed the first human dosimetric experience with [^89^Zr]Zr-panitumumab in three patients with metastatic colon cancer (Figure 2) [31]. They calculated the effective dose within range of extrapolated estimates from preclinical studies with reasonable safe and dosimetry for clinical imaging [31] According to our knowledge, no clinical studies about [^89^Zr]-panitumumab have been published yet.

### 2.2. HER2

The Human Epidermal Growth Factor Receptor 2 (HER2), or HER2 receptor, is a transmembrane receptor tyrosine kinase that plays a crucial role in regulating cell growth, proliferation, differentiation, and survival. Unlike other members of the ErbB family, the HER2 does not directly bind to ligands. Its activation results from heterodimerization with another ERBB member or by homodimerization when HER2 concentration is high, for instance, in cancer [32]. The HER2 receptor is characterized by oncogenic power [33]. It is one of the molecular markers of ductal breast cancer, but at the same time, it is overexpressed in other adenocarcinomas, such as gastric cancer [34]. The detection of its gene amplification is associated with a worst prognosis but is also the major criterion for sensitivity selection to treatment with the anti-HER2 mAb trastuzumab, also called Herceptin [35,36]. Other drugs targeting HER2 are represented by pertuzumab, lapatinib, neratinib, tucatinib and T-DM1, or ado-trastuzumab emtansine and fam-trastuzumab deruxtecan [33].

The first human study with [^64^Cu]Cu-DOTA (1,4,7,10-tetraazacyclododecane-1,4,7,10-tetraacetic acid) (^64^Cu)-trastuzumab was conducted by Tamura et al. in a cohort of six patients with primary or metastatic HER2-positive breast cancer [37]. They demonstrated the successful visualization of HER2-positive primary breast carcinoma and metastatic lesions, including cerebral metastases. The detection of brain metastases is very controversial. On one hand, this technique could help the clinicians to detect HER2 density in metastatic sites where it is not possible to obtain a surgery sample, but on the other hand, the trastuzumab penetration is possible only with a disrupted blood–brain barrier. Moreover, they highlighted more difficulties in the detection of pathological tissue in strictly adjacency of nonspecific high-uptake sites such as heart and liver. Cold trastuzumab assumption may not have influenced the results.

Later, Carrasquillo et al. [38] examined the use of [^64^Cu]Cu-trastuzumab in patients with metastatic HER2-positive breast cancer. Their findings were very divergent from the previous study. They did not visualize uptake in the primary tumor, relatable with chronic assumption of “cold” trastuzumab and consequent binding of cell receptors. Another major difference with previous cohort was the dose (5 mg versus 86 microgram). In accordance to the shorter half-life of [^64^Cu] (equal to 13 h), Carrasquillo et al. performed acquisitions after 1 and 24 h of radiolabeled injection [38], while Tamura et al. chose the protocol after 48 h after injection [37].

In a cohort of patients with biopsy-confirmed HER2-positive metastatic breast cancer and no anti-HER2 therapy for ≥4 months, Mortimer et al. [39] compared the detection sensitivity of [^64^Cu]Cu-trastuzumab images at 24 and 48 h, resulting, respectively, in detections of 77% and 89%. Tumor uptake was substantially stable between 24 h and 48 h, with only a modest increase, while lymph node detection in the neck, upper thorax and mediastinum remained difficult at 24 h due to masking by the adjacent blood pool and liver, but increased by 48 h. The uptake appeared highly variable between and within patients, with a consequent possibility to foresee responders from non-responders.

More recently, Mortimer et al. [40] demonstrated a positive association between the response to trastuzumab–emtansine (T-DM1) and tumor uptake of [^64^Cu]Cu-trastuzumab, measured as SUVmax. In fact, T-DM1-responsive patients had higher uptake than nonresponsive patients both at day 1 and day 2 after injection. Moreover, patients with a day 2 high SUVmax had a median time to treatment failure significantly longer than patients with low SUVmax.

The use of trastuzumab labeled with [^89^Zr] is an alternative to assess HER2 status during the disease [41]. Due to its long half-life, it is characterized by less favorable dosimetry, as well as by the property to perform a greater number of scans over time. The first study to evaluate the optimal dosage and time of administration of [^89^Zr]Zr-trastuzumab was conducted by Dijkers et al. [41]. Firstly, they evaluated the physiological distribution of the tracer and then the relative uptake value (RUV) to quantify [^89^Zr]Zr-trastuzumab distribution. RUV is a semiquantitative representation of the tissue-to-background ratio, related to the amount of tracer present in the body during the scan and independent of the rate of excretion.

The possibility to evaluate HER2 status in vivo through [^89^Zr]Zr-trastuzumab PET/CT to support clinical choice and treatment strategy was also supported by Bensch et al. [42]. In HER2 PET evaluation, the physiological liver uptake appears to be a limitation of the method. The published studies have a very heterogeneous inclusion criteria of population, as they have involved both naive trastuzumab patients and patients who were currently taking this therapy without a standard dosage. The reduced visualization of hepatic tissue could be partially resolved by administering 45 mg of cold trastuzumab before PET scan [40] or by performing the examination after a standard therapeutic dose of trastuzumab [39]. As in other ImmunoPET tracers labeled with [^89^Zr], the best timing to evaluate tumor uptake of [^89^Zr]Zr-trastuzumab was 4–5 days post injection [43].

In breast cancer, [^89^Zr]Zr-trastuzumab is also used to monitor the alteration of antigen expression in the treatment response of novel anti-cancer agents. In particular, HSP90 (heat shock protein 90) is a molecular chaperone which plays a critical role in the protein folding and function for a broad range of client proteins, depending on the HER2 expression. Gaykema et al. [44] determined the degradation of HER2 caused by the novel HSP90 inhibitor NVP-AUY922. Two [^89^Zr]Zr-trastuzumab PET scans were performed at baseline and at day 15 after treatment with HSP90 inhibitor NVP-AUY922. The heterogeneous uptake was positively correlated with CT responses in individual lesions. Also, in this cohort, they confirmed heterogeneous SUVmax values, both intra-patient and inter-patient, in line with the heterogeneity of tumor responses at CT scan. In the same study, the authors tested [^89^Zr]Zr-bevacizumab (which we deal with in the next paragraph) but without significant correlation.

Tumor heterogeneity and, consequently, the different prevision of treatment response, was also studied during the ZEPHIR trial by Gebhart et al. [45]. Enrolled breast cancer patients were evaluated both with [^89^Zr]Zr-trastuzumab PET/CT at baseline and with 2-Deoxy-2-[^18^F]fluoroglucose (2-[^18^F]FDG) PET/CT at baseline and after one cycle of trastuzumab emtansine. The authors combined results of the two different PET scans in order to predict response to trastuzumab-emtansine treatment. “Concordant” patients were defined as positive [^89^Zr]Zr-trastuzumab and FDG early responders, or negative [^89^Zr]Zr-trastuzumab and FDG early non-responders. In these groups, negative and positive predictive values to predict response to treatment were 100%, associated with significantly different times to treatment failure (15 vs. 2.8 months). “Discordant patients” (positive [^89^Zr]-trastuzumab and FDG early non-responders, or negative [^89^Zr]Zr-trastuzumab and FDG early responders) demonstrated how HER2 PET can evaluate the presence of the treatment target but not eventually an intrinsic resistance phenomenon.

Finally, Jauw et al. combined the abovementioned studies [26,42] in association with the Dutch Trial Register to confirm the interobserver reproducibility [46]. The SUVmax, SUVpeak and SUVmean results were excellent with variability equal to 0% through manual procedure. Regarding semi-automatic delineation, the results were not robust, probably due to the noted heterogeneous and lower tumor/background ratio uptake.

Pertuzumab is a new HER2 mAb that binds in a distinct site from trastuzumab and appears to be more efficient than it [47]. Ulaner et al. were the first ones to show promising results about the use of [^89^Zr]Zr-pertuzumab in a cohort of women with biopsy-proven HER2-positive invasive ductal breast cancer. In this first-in-human trial, they assessed the safety and successful HER2-targeted imaging with promising results for the future [48].

**Figure 3:**
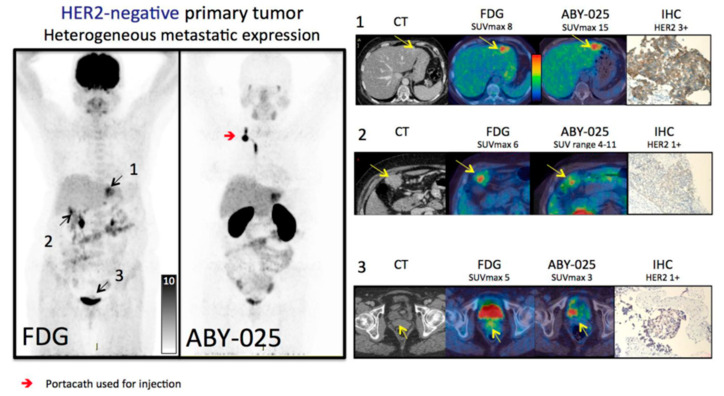
Multipanel image comparing [^68^Ga]Ga-ABY-025 PET/CT and 2-[^18^F]FDG (2-Deoxy-2-[^18^F]fluoroglucose) PET/CT in a metastatic breast cancer patient with an HER2-negative primary tumor. 2-[^18^F]FDG-PET/CT showed metastases in left liver lobe, peritoneal lymph nodes and cervix of uterus, while ABY-025 uptake was high in the liver lesion, low in peritoneal metastases and absent in the cervix. At IHC, the liver lesions confirmed true metastases, whereas the other sites were true negative. Modified from Sörensen J et al. [50] published under a Creative Commons Attribution (CC BY-NC) License https://creativecommons.org/licenses/by-nc/4.0/ (last access: 26 November 2023).

In the literature, HER2 status in breast cancer has been evaluated with a radiolabeled single-domain antibody called Nanobody. Keyaerts et al. assessed the safety, biodistribution and tumor uptake of [^68^Ga]Ga-HER2-Nanobody for the first time in human subjects [49]. The affibody molecule ABY-025 labeled with [^68^Ga] showed the capability to discriminate HER2-positive metastases and a positive correlation between SUV and HER2 expression. They showed high uptake in almost all metastatic sites but variable uptake in primary lesions. Based on the uptake decrease in liver parenchyma, the authors defined 90 min post-injection as the optimal time point for signal-to-noise ratio image acquisition. Later time points were not assessable due to the short half-life of [^68^Ga]. These findings were also confirmed by Sörensen et al., with significant correlation between SUV and HER2 scores at biopsy (Figure 3) and five-times-higher uptake in HER2-positive than in HER2-negative lesions [50]. In order to improve the discriminative capability of HER2 imaging using ABY-025 in breast cancer metastases, Sandberg et al. [51] found the spleen as the best reference tissue, followed by blood pool and lung. In particular, the tumor-to-spleen ratio was highly correlated to SUV in metastases after 2 h and reached the accuracy of 100% for detecting IHC HER2 status at 4 h after injection.

[^89^Zr]Zr-trastuzumab PET was also performed in other type of cancers. In particular, in esophagogastric (EG) cancer, O’Donoghue [52] first assessed the feasibility of optimal imaging and confirmed the optimal scan time at 5–8 days after administration. Moreover, Sanchez-Vega et al. [53] evaluated the predictive role of this radiotracer in response to afatinib. Afatinib is an irreversible pan-HER kinase inhibitor and usually administered in HER2-positive EG cancer. Their results suggested that homogenous pre-treatment [^89^Zr]Zr-trastuzumab uptake may be a marker of afatinib sensitivity and, consequently, the heterogeneous uptake at baseline may be an indicator of a poor response.

### 2.3. HER3

HER3, also member of the ErbB/HER receptor tyrosine kinase (RTK) family, was initially considered to be inactive, but studies have shown that HER3 contains weak kinase activity and acts as an obligate allosteric activator [54,55]. It is widely expressed in human adult tissues and is known to preferentially dimerize with other ErbB family members, especially HER2. HER3 is involved in the activation of downstream signaling pathways, including the PI3K/Akt pathway, and is associated with worse survival in various solid tumors, particularly when HER2 is overexpressed. It has also been implicated in tumorigenesis, cancer progression and targeted therapy resistance in several types of cancer [56] Among the major therapeutic representatives targeting HER3, we can find mAbs such as lumretuzumab, patritumab and zenocutuzumab and small molecules like lapatinib, erlotinib, gefitinib, afatinib and neratinib [57,58,59,60].

GSK2849330 is an anti-HER3 monoclonal antibody that has been studied in the context of HER3-expressing solid tumors. It has been evaluated in a phase I, first-in-human, open-label study to assess its safety, pharmacokinetics, pharmacodynamics and preliminary activity in patients with HER3-expressing solid tumors [61,62]. Its mechanisms of action are through an antibody-dependent cell-mediated cytotoxicity (ADCC) and complement dependent cytotoxicity (CDC) [62].

Menke-van der Houven van Oordt et al. [63] described the biodistribution and dose-receptor occupancy relationship of GSK2849330 labeled with [^89^Zr] in patients with advanced HER3-expressing solid tumors. In the same cohort of patients, they compared the PET scans after two different dose tracer administration at baseline at 1 day and after 14 days. At 14 days, to evaluate the dose-dependent inhibition, the authors also administered a variable total 24–30 mg/kg dose of unlabeled mAb. So, they observed a reduced accumulation of radiolabeled mAb in tumor lesions after treatment, defined by SUV. With the same scheme of scans, through the administration of a lumretuzumab dose for saturation analysis (400, 800 or 1600 mg) and [^89^Zr]Zr-lumretuzumab as a PET tracer, Bensch et al. [42] did not confirm the HER3 binding saturation with increasing mass doses of mAb.

Lumretuzumab (RG7116, RO5479599) is a glycoengineered humanized mAb directed against the extracellular domain of HER3 but binds a different epitope than GSK2849330 [63]. Similar results on the heterogeneity of tumor uptake were also reported in this study.

An another HER3 mAb is patritumab (U3-1287, AMG 888), which promotes receptor internalization, leading to the inhibition of basal and ligand-induced activation and downstream signaling [64,65] Craig Lockhart et al. [66] evaluated the safety, dosimetry and apparent receptor occupancy of [^64^Cu]Cu-DOTA-patritumab in patients with advanced solid tumors. As in the abovementioned studies, patients performed two PET/CT scans, one at baseline and the other at 9 days after infusion of unlabeled patritumab (9.0 mg/kg). The results assessed the reduction of TBR uptake between the two imaging analyses, which was probably related to receptor occupancy.

### 2.4. VEGF

The vascular endothelial growth factor, or VEGF, and one of its related receptors (VEGF receptor 2 (VEGFR-2)), are the most prominent regulators of angiogenesis and have been implicated as key drivers of tumor vascularization. The main stimulus for its increased expression is hypoxia [67,68]. It is secreted by various cell types, including endothelial cells, macrophages and platelets, and acts on endothelial cells, the cells lining the inner surface of blood vessels. VEGF binds to its specific receptor triggering a cascade of signaling events that promote angiogenesis. In cancer, tumor cells often exhibit increased VEGF expression, leading to excessive angiogenesis and tumor vascularization. This enhanced blood supply provides tumors with nutrients and oxygen, facilitating tumor growth and metastasis. VEGF/VEGFR signaling also promotes tumor cell invasion and survival. The most important anti-VEGF/VEGFR representative drugs are bevacizumab, ramucirumab, pazopanib, sunitinib, sorafenib, lenvatinib, and axitinib [69].

Bevacizumab (Avastin^®^) is a mAb-targeting vascular endothelial growth factor A (VEGF-A). It exerts its activity by neutralizing VEGF and blocking its signal transduction through both the VEGFR-1 and VEGFR-2 receptors, thereby inhibiting VEGF-induced cell proliferation, survival, permeability, nitric oxide production, migration and tissue factor production [70]. Bevacizumab has been approved for the treatment of various cancers, including metastatic colorectal cancer (mCRC), metastatic breast cancer, non-small-cell lung cancer, glioblastoma, metastatic renal cell carcinoma (mRCC), ovarian cancer and cervical cancer [71].

The first clinical feasibility study with [^89^Zr]Zr-bevacizumab PET/CT in breast cancer patients was conducted by Gaykema et al. al [72]. The [^89^Zr]Zr-bevacizumab uptake was visualized in 96.1% of primary lesions with a significant correlation between [^89^Zr]Zr-bevacizumab tumor uptake and VEGF-levels as measured by ELISA (enzyme-linked immunosorbent assay).

The role of [^89^Zr]Zr-bevacizumab was also tested in NSCLC, and the first pilot study was performed by Bahce I.et al. [73]. In a cohort of NSCLC at stage IV, the authors confirmed that all pathological lesions, both tumor and metastases, had four-times higher uptake compared to non-tumor tissues, both at 4 and 7 days after injection. Lymph nodes and metastases had greater uptake than tumors, probably due to breathing movement-induced and partial volume effects on primary lesions. Moreover, they evidenced a positive but not significant correlation between SUVpeak and OS (overall survival) or PFS (progression-free survival).

**Figure 4:**
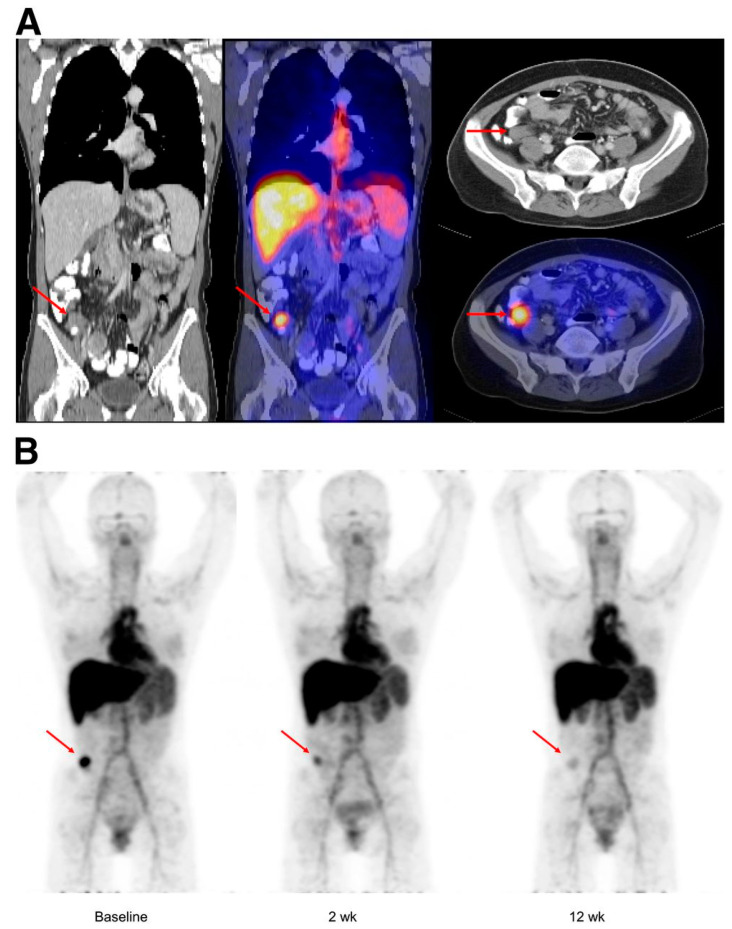
[^89^Zr]Zr-bevacizumab PET images in patient with metastatic midgut carcinoid obtained 4 days post-injection. Upper panel (**A**) includes low-dose CT and fusion PET/CT images at the level of the abdominal tumor lesion (red arrows). Lower panel (**B**) illustrates MIP images obtained at baseline, 2 weeks and 12 weeks after everolimus treatment, proving a progressive decrease on the tumor uptake (red arrows). Note the physiologic [^89^Zr]Zr-bevacizumab uptake in the heart, liver, spleen and blood pool. Reproduced from van Asselt SJ et al. [75] for non-commercial use.

Similar results were found with [^89^Zr]Zr-bevacizumab in both metastatic mRCC and neuroendocrine tumor (NET) patients [74,75]. In both studies, [^89^Zr]Zr-bevacizumab uptake was very heterogeneous both in intra-patient and inter-patient evaluations at baseline (Figure 4). After therapy with bevacizumab/IFNa in mRCC and with everolimus in NET, significant reduction of tracer activity was demonstrated. Consistently with previous studies, these latter studies showed a strong correlation between uptake, and prognosis was confirmed.

## 3. Conclusions

ImmunoPET combines the advantages of in vivo and non-invasive immunohistochemistry and the strengths of PET/CT, with growing role in the clinical management. These preliminary studies support the potential of ImmunoPET targeting RTKs as future imaging biomarker with promising results regarding the patients’ selection, drug effect evaluation and prognostic role. Thus, to successfully introduce ImmunoPET tracers into the daily clinical practice validation of these reported results, it is necessary to perform further prospective trials with enlarged sample sizes with the most promising ImmunoPET radiotracers.

## Figures and Tables

**Table 1:** Summary of the ongoing clinical trials investigating ImmunoPET targeting RTK (source: https://clinicaltrials.gov/), accessed up to 21 November 2023.

ImmunoPET Tracer	Status	Identifier Number	Phase	Study Type	Target	Study Title	Conditions	Country	Last Update
[^89^Zr]Zr-DFO-nimotuzumab	Recruiting	NCT04235114	I, II	Interventional	EGFR	Evaluation of [^89^Zr]Zr-DFO-nimotuzumab for Non-invasive Imaging of EGFR+ Cancers by Positron Emission Tomography (PET)	Lung Cancer Colorectal Cancer	Saskatchewan, Canada	2022-05
[^89^Zr]Zr-panitumumab	Recruiting	NCT05747625	I	Interventional	EGFR	([^89^Zr]Zr-Panitumumab) With PET/CT for Diagnosing Metastases in Patients With Head and Neck Squamous Cell Carcinoma	Head and Neck Squamous Cell Carcinoma Metastatic Head and Neck Squamous Cell Carcinoma Stage IV Cutaneous Squamous Cell Carcinoma of the Head and Neck	Nashville, USA	2023-07
[^89^Zr]Zr-panitumumab	Not yet recruiting	NCT05423197	II	Interventional	EGFR	Study Evaluating Zr-Panitumumab for Assessment of Suspected Metastatic Lesions on 2-[^18^F]FDG-PET/CT in Head and Neck Squamous Cell Carcinoma	Head and Neck Squamous Cell Carcinoma	San Francisco, USA	2023-09
[^89^Zr]Zr-Panitumumab	Recruiting	NCT05183048	Early Phase I	Interventional	EGFR	Comparison of [^89^Zr]Zr-Panitumumab and [^18^F]-Fluorodeoxyglucose to Identify Head and Neck Squamous Cell Carcinoma	Head and Neck Squamous Cell Carcinoma	Birmingham, USA	2023-10
[^64^Cu]Cu-DOTA-trastuzumab	Active, not recruiting	NCT01093612	NA	Interventional	HER2	Positron Emission Tomography in Women With Advanced HER2-Positive Breast Cancer	Breast Cancer Stage IV Breast Cancer	Duarte, USA	2023-03
[^64^Cu]Cu-DOTA-trastuzumab	Active, not recruiting	NCT02226276	NA	Interventional	HER2	Copper Cu 64-DOTA-Trastuzumab PET in Predicting Response to Treatment With Ado-Trastuzumab Emtansine in Patients With Metastatic HER2 Positive Breast Cancer	Bone Metastases, HER2-positive Breast Cancer, Liver Metastases, Lung Metastases, Recurrent Breast Cancer, Soft Tissue Metastases, Stage IV Breast Cancer	Duarte, USA	2023-02
[^64^Cu]Cu-DOTA-Trastuzumab	Active, not recruiting	NCT02827877	II	Interventional	HER2	[^64^Cu]Cu-DOTA-trastuzumab PET and Markers Predicting Response to Neoadjuvant Trastuzumab + Pertuzum in HER2+ Breast Cancer	HER2-Positive Breast Carcinoma, Stage IIIA Breast Cancer, Stage IIIB Breast Cancer, Stage IIIC Breast Cancer	Duarte, USA	2023-08
[^89^Zr]Zr-DFO*-Trastuzumab	Recruiting	NCT05955833	I	Interventional	HER2	[^89^Zr]-DFO*-Trastuzumab PET in Patients With Gastric or Breast Cancer—a Pilot Study (HER Image)	Breast Cancer, Metastatic Breast Cancer, HER2-Positive Breast Cancer, Gastric Cancer, Metastatic Gastric Cancer, HER2-Positive Gastric Cancer	Amsterdam, The Netherlands	2023-07
[^64^Cu]Cu-DOTA-trastuzumab	Recruiting	NCT05376878	IV	Interventional	HER2	An Investigational Scan ([^64^Cu]Cu-DOTA-Trastuzumab PET/MRI) in Imaging Patients With HER2+ Breast Cancer With Brain Metastasis	Anatomic Stage IV Breast Cancer, AJCC v8 Metastatic Breast Carcinoma, Metastatic Malignant Neoplasm in the Brain	Duarte, USA	2023-02
[^89^Zr]Zr-Trastuzumab	Recruiting	NCT03321045	Early Phase I	Interventional	HER2	Positron Emission Tomography (PET) Imaging With Zirconium-89 (^89^Zr)-Trastuzumab for Prediction of HER2 Targeted Therapy Effectiveness	Breast Cancer	Birmingham, USA	2023-02
[^64^Cu]Cu-DOTA-trastuzumab	Active, not recruiting	NCT01939275	NA	Interventional	HER2	[^64^Cu]Cu-DOTA-Trastuzumab PET/CT in Studying Patients With Gastric Cancer	Adenocarcinoma of the Gastroesophageal Junction, Diffuse Adenocarcinoma of the Stomach, Intestinal Adenocarcinoma of the Stomach, Mixed Adenocarcinoma of the Stomach, Stage IA-IV Gastric cancer	Duarte, USA	2023-05
[^89^Zr]Zr-trastuzumab	Active, not recruiting	NCT01565200	II	Interventional	HER2	HER2 Imaging Study to Identify HER2 Positive Metastatic Breast Cancer Patient Unlikely to Benefit From T-DM1 (ZEPHIR)	HER-2-Positive Breast Cancer	Brussels, Belgium	2023-08
[^68^Ga]Ga-HER2-affibody	Recruiting	NCT04769050	NA	Observational	HER2	Dynamic Observational Study With PET of [^68^Ga]Ga-HER2-affibody in Anti-HER2 Treatment	Breast Cancer	Shanghai, China	2022-04
[^68^Ga]Ga-HER2-affibody	Recruiting	NCT04281641	NA	Observational	HER2	Markers to Evaluate the Efficacy of PH-based Regimen as a Neoadjuvant Therapy for Operable HER2 Positive Breast Cancer (PHC-BC)		Shanghai, China	2020-05
[^18^F]F-GE-226	Recruiting	NCT03827317	NA	Interventional	HER2	HERPET-A Novel PET Imaging Study of HER2 in Breast Cancer	Breast Cancer	London, UK	2023-01
[^89^Zr]Zr-ss-pertuzumab	Recruiting	NCT04692831	I	Interventional	HER2	Testing a New Imaging Agent to Identify Cancer	HER-2-Positive Malignant Carcinoma, of Breast HER-2 Protein Overexpression, HER2-Positive Metastatic Breast Cancer	New York, Newport Beach, USA	2023-11
[^68^Ga]Ga-ABY-025	Recruiting	NCT05619016	II	Interventional	HER2	[^68^Ga]Ga-ABY-025 PET for Quantification of HER2-status in Solid Tumors	Esophageal Neoplasms, Gastric Neoplasms, Malignant Breast Cancer, HER2-Positive Gastric Cancer	Stockholm, Sweden	2022-11
[^18^F]F-HER2	Not yet recruiting	NCT05983796	NA	Interventional	HER2	[^18^F]F-HER2 PET in Evaluating the Efficacy of Anti-HER2 Therapy for Urothelial Carcinoma.	Urothelial Carcinoma	Hangzhou, China	2023-08
[^68^Ga]Ga-GaNOTA-Anti-HER2 VHH1	Recruiting	NCT03924466	II	Interventional	HER2	Repeatability of [^68^Ga]Ga-NOTA-Anti-HER2 VHH1 PET/CT in Breast Carcinoma Patients (VUBAR)	Metastatic Breast Carcinoma, Locally Advanced Breast Cancer, Cancer of Pancreas, Solid Tumor With Intermediate or High HER2 Expression, Salivary Gland Cancer, Gastric Cancer, Endometrial Cancer, Uterine Cancer, Non Small Cell Lung Cancer, Biliary Tract Cancer, Cholangiocarcinoma, Colorectal Cancer, Urothelial Carcinoma, Prostate Cancer	Brussels, Belgium	2023-01
[^68^Ga]Ga-ABY-025	Recruiting	NCT03655353	II/III	Interventional	HER2	A Study of [^68^Ga]Ga-ABY-025 PET for Non-invasive Quantification of HER2-expression in Advanced Breast Cancer (Affibody-3)	HER2-positive Breast Cancer	Uppsala, Sweden	2021-09
[^68^Ga]Ga-HER2-Affibody, [^18^F]-FDG	Recruiting	NCT04758416	II	Observational	HER2	Study on the Value of Non-invasive Dual-Pet Information in Subtype of Metastatic Breast Cancer	Breast Cancer	Shanghai, China	2022-04
[^68^Ga]Ga-HER2 Affibody	Recruiting	NCT05535621	NA	Observational	HER2	[^68^Ga]Ga-HER2 Affibody PET/CT Imaging for HER2-Positive Cancer Patients	HER2-Positive Cancer	Wuhan, China	2023-02
[^68^Ga]Ga-NOTA-Anti-HER2 VHH1	Recruiting	NCT03331601	II	Interventional	HER2	Evaluation of [^68^Ga]Ga-NOTA-Anti-HER2 VHH1 Uptake in Brain Metastasis of Breast Carcinoma Patients	Breast Neoplasm, Breast Carcinoma Receptor, ErbB-2	Brussels, Belgium	2023-07
[^68^Ga]/[^18^F]-HER2 Affibody	Recruiting	NCT04547309	NA	Interventional	HER2	Research for the Molecular Imaging of the HER2 Targeting Tracer	HER2-Positive or Suspicious Positive Tumors	Beijing, China	2023-11
[^68^Ga]Ga-HER2 Affibody	Not yet recruiting	NCT05411432	Early Phase I	Interventional	HER2	Clinical Study of [^68^Ga]-Labeled HER2 Affibody Analogues	HER2-Positive Breast Cancer and Gastric Cancer	Xijing, China	2022-06
[^18^F]F-ISO-1	Not yet recruiting	NCT02284919	Phase I	Interventional	HER2	[^18^F]F-ISO-1 Positron Emission Tomography (PET/CT) in Primary Breast Cancer (ISO-1Primary)	Breast Cancer	Philadelphia, USA	2023-01
[^68^Ga]/[^131^I] SGMIB-5F7	Recruiting	NCT05982626	NA	Interventional	HER2	Study of [^68^Ga]/[^131^I] SGMIB-5F7 PET Imaging Targeting HER2-positive in the Diagnosis of Metastatic Breast Cancer	HER2-Positive Breast Cancer	Shanghai, China	2023-11
[^18^F]F-GEH121224	Not yet recruiting	NCT05634954	I	Interventional	HER2	Study to Evaluate Safety and Dosimetry of [^18^F]F-GEH121224 in Patients With Locally Advanced or Metastatic Breast Cancer	Breast Cancer	Houston, USA	2023-09
[^89^Zr]Zr-Bevacizumab	Recruiting	NCT05685836	NA	Observational	VEGF	[^89^Zr]Zr-Bevacizumab PET/CT Imaging in NF2 Patients	Neurofibromatosis 2	Leiden, The Netherlands	2023-01

Notes: 2-[^18^F]FDG, 2-Deoxy-2-[^18^F]fluoroglucose; EGFR, epidermal growth factor receptor; NA, not applicable; VEGF, vascular endothelial growth factor.

## Data Availability

Data sharing not applicable.
